# Association Between Chronic Kidney Disease and Incident Ménière’s Disease Using 16 Years of Nationwide Longitudinal Data

**DOI:** 10.3390/jcm15187328

**Published:** 2026-09-21

**Authors:** Eun Soo Kim, Jwa-Kyung Kim, Heejin Kim, Joong Seob Lee, Jeong Wook Kang, Min-Jeong Kim, Ho Suk Kang, Dae Myoung Yoo, Kyeong Min Han, Nan Young Kim, Hyo Geun Choi, Mi Jung Kwon

**Affiliations:** 1 Department of Radiology, Hallym University Sacred Heart Hospital, Hallym University College of Medicine, Anyang 14068, Republic of Korea; silwater007@hallym.or.kr (E.S.K.); drkmj@hallym.or.kr (M.-J.K.); 2 Division of Nephrology, Department of Internal Medicine, Hallym University Sacred Heart Hospital, Hallym University College of Medicine, Anyang 14068, Republic of Korea; kjk816@hallym.or.kr; 3 Department of Otorhinolaryngology-Head & Neck Surgery, Hallym University Sacred Heart Hospital, Anyang 14068, Republic of Korea; heejin5020@hanmail.net (H.K.); entkang@hallym.or.kr (J.W.K.); 4 Division of Gastroenterology, Department of Internal Medicine, Hallym University Sacred Heart Hospital, Hallym University College of Medicine, Anyang 14068, Republic of Korea; putamenn@hanmail.net; 5 Hallym Data Science Laboratory, Hallym University College of Medicine, Anyang 14068, Republic of Korea; ydm1285@naver.com (D.M.Y.); km_han@hallym.ac.kr (K.M.H.); 6 Hallym Institute of Translational Genomics and Bioinformatics, Hallym University Medical Center, Anyang 14068, Republic of Korea; honeyny78@gmail.com; 7 Suseo Seoul E.N.T. Clinic, 10, Bamgogae-ro 1-gil, Gangnam-gu, Seoul 06349, Republic of Korea; mdanalytics@naver.com; 8 Department of Pathology, Hallym University Sacred Heart Hospital, Hallym University College of Medicine, Anyang 14068, Republic of Korea

**Keywords:** chronic kidney disease, Ménière’s disease, epidemiology, cohort study, propensity score, vestibular disorder

## Abstract

**Background:** Although chronic kidney disease (CKD) has been associated with several forms of auditory and vestibular impairment, its relationship with Ménière’s disease (MD) has not been well established at the population level. We investigated whether CKD is associated with subsequent development of MD in a large national cohort. **Methods:** Data were obtained from the Korean National Health Insurance Service–Health Screening Cohort for 2002–2019. The analysis included 15,943 participants with CKD and 63,772 comparison participants matched at a 1:4 ratio according to age, sex, household income, and region of residence. Propensity-score overlap weighting was additionally applied to balance measured baseline characteristics. Cox proportional hazards models were used to estimate hazard ratios (HRs) and 95% confidence intervals (CIs) for incident MD. **Results:** During 67,471 person–years of follow-up in the CKD group and 330,701 person–years in the comparison group, 173 and 903 participants, respectively, developed MD. The crude HR was 0.92 (95% CI, 0.79–1.09; *p* = 0.346), and the overlap-weighted point estimate was 0.95 (95% CI, 0.84–1.07; *p* = 0.414). **Conclusions:** The available point estimates did not indicate a higher hazard of incident MD among participants with CKD, but the findings should be interpreted cautiously because weighted variance estimation could not be evaluated and death was not modeled as a competing event. Further studies with prospectively retained individual-level data and competing-risk analyses are warranted.

## 1. Introduction

Ménière’s disease (MD) is an episodic inner-ear disorder characterized by recurrent vertigo accompanied by fluctuating sensorineural hearing impairment, tinnitus, and aural pressure or fullness [1]. Recurrent attacks and progressive auditory dysfunction can substantially impair daily functioning and quality of life [1,2]. In addition to this physical burden, patients with vestibular disorders, including MD, may experience substantial psychological morbidity, with depressive and anxiety-related symptoms contributing further to impaired quality of life [3]. Nevertheless, estimates of MD occurrence differ considerably across populations, partly because large-scale epidemiologic data remain limited [2]. In Japan, reported prevalence estimates range from 5.8 to 37 per 100,000 adults [4]. In Korea, the annual incidence was reported to increase from 30.02 per 100,000 in 2013 to 118.48 per 100,000 in 2017, potentially reflecting population aging, lifestyle changes, and greater recognition of the disease [5].

The biological processes responsible for MD have not been fully established. Proposed contributors include immune dysregulation, viral triggers, genetic susceptibility, and impaired regulation of endolymphatic volume or absorption [1,6]. Chronic kidney disease (CKD) is characterized by persistent impairment of renal structure or function and is accompanied by systemic abnormalities including chronic inflammation, endothelial injury, uremic toxin accumulation, oxidative stress, microvascular dysfunction, and disturbances of fluid and electrolyte homeostasis [6,7,8,9,10,11,12]. Electrolyte regulation is particularly relevant to inner-ear physiology because maintenance of the ionic composition of endolymph and perilymph is essential for normal cochlear and vestibular function. A recent clinical study further suggested that variations in serum sodium and potassium levels may be associated with sensorineural hearing loss and tinnitus [13]. These observations provide an additional biological rationale for investigating whether CKD-related systemic and electrolyte disturbances influence inner-ear disorders.

The auditory and vestibular organs may be particularly susceptible to systemic vascular disturbances because the labyrinth is supplied by the labyrinthine artery, which has little or no effective collateral circulation [14]. Within the cochlea, the stria vascularis is a metabolically active and richly perfused structure responsible for generating the electrochemical environment required for normal auditory transduction [12,15]. Renal and cochlear tissues also share several physiological features. The stria vascularis, renal glomerular structures, and tubular epithelium depend on tightly regulated ion transport, specialized epithelial barriers, and microvascular homeostasis [11,12,16]. These shared characteristics have raised the possibility that disorders affecting renal function may also contribute to auditory or vestibular abnormalities [11,12,16].

Epidemiologic observations provide some support for a kidney–inner ear relationship. CKD has been associated with sensorineural hearing impairment, tinnitus, and abnormalities of balance in several clinical and population-based investigations [11,16,17,18,19]. A cross-sectional analysis from China involving 3762 participants, for example, found independent relationships between CKD and both hearing impairment and tinnitus, with more pronounced associations among participants with advanced renal disease [20]. In another study of 88 Indian patients with CKD receiving hemodialysis, 78.4% reported tinnitus, vertigo, or a combination of the two symptoms [18]. Such findings, however, concern auditory or vestibular dysfunction broadly and do not establish whether CKD predisposes specifically to MD. Evidence evaluating incident MD at the population level remains scarce, and the available findings have not demonstrated a consistent relationship [17]. A previous Korean nationwide cohort study reported an association between CKD and MD, although the magnitude of the relationship was attenuated after adjustment for potential confounders [15]. Important uncertainties therefore remain regarding whether CKD itself independently contributes to MD or whether the observed relationship is explained by accompanying demographic, metabolic, behavioral, or clinical characteristics.

The present study re-evaluated this association using the Korean National Health Insurance Service–Health Screening Cohort with follow-up through 2019. In contrast to analyses based primarily on claims-based demographic and comorbidity information, the health-screening database enabled incorporation of lifestyle variables and directly measured metabolic indicators. We additionally applied 1:4 matching and propensity-score overlap weighting and used a more restrictive MD definition requiring repeated diagnostic claims together with audiometric testing. Our primary objective was to determine whether CKD is independently associated with incident MD during long-term follow-up.

## 2. Materials and Methods

### 2.1. Data Source and Study Framework

We performed a retrospective longitudinal cohort analysis using the Korean National Health Insurance Service–Health Screening Cohort (NHIS-HEALS). The database contains information on 514,866 adults aged 40–79 years at cohort enrollment who were sampled from individuals participating in the national health-screening program. Approximately 10% of eligible health-screening participants were randomly selected for inclusion at baseline [21].

The database links health-screening measurements with longitudinal insurance claims recorded between 2002 and 2019. Diagnoses are coded according to the International Classification of Diseases, 10th Revision (ICD-10), whereas medical procedures and examinations are recorded using national insurance claim codes [21]. Because virtually all Korean residents are covered by the National Health Insurance Service, the database permits longitudinal assessment of healthcare utilization in a large population. This study was based exclusively on de-identified human health-screening and administrative claims data. No animals, tissue specimens, histological sections, ultrastructural assessments, or microscopy-based image analyses were included; therefore, blinding and section/field sampling procedures applicable to experimental pathology studies were not relevant to this study.

The dataset provided for research was fully anonymized and curated by the Korean government. The study protocol complied with the principles of the Declaration of Helsinki and was approved by the Institutional Review Board of Hallym University (IRB No. 2022-10-008; approval date: 22 October 2022). The requirement for individual informed consent was waived because the study used de-identified secondary data.

### 2.2. Ascertainment of CKD and Ménière’s Disease

CKD served as the exposure of interest. Participants were classified as having CKD when they had at least two claims carrying ICD-10 code N18 or N19 and/or a record of dialysis identified using procedure codes O7010, O7020, or O7070 [22]. Requiring repeated claims was intended to reduce misclassification resulting from isolated or provisional diagnostic coding.

Incident MD constituted the primary outcome. According to the Bárány Society criteria, definite MD requires at least two spontaneous episodes of vertigo lasting 20 min to 12 h, audiometrically documented low- to medium-frequency sensorineural hearing loss in the affected ear, fluctuating aural symptoms (hearing change, tinnitus, or fullness) in the affected ear, and exclusion of a more appropriate vestibular diagnosis [23]. Probable MD is defined more broadly by episodic vestibular symptoms lasting 20 min to 24 h together with fluctuating aural symptoms [23]. Because the present study used administrative claims rather than individual clinical records, symptom duration and all components of the Bárány criteria could not be directly adjudicated. Therefore, to improve diagnostic specificity, MD was operationally identified using ICD-10 code H81.0 and required at least two diagnostic claims together with an audiometric examination coded as E6931–E6937 or F6341–F6348 [24].

### 2.3. Study Population and Matching

Participant selection began with all 514,866 individuals in the health-screening cohort. Among them, 17,478 initially satisfied the claims-based criteria potentially indicating CKD. Several exclusions were subsequently applied to establish a cohort suitable for evaluating newly recognized CKD and subsequent MD.

Participants were excluded when CKD had already been recorded in 2002, when only one CKD diagnostic claim was available, or when required health-screening measurements were unavailable. Baseline variables required for analysis included body mass index (BMI), blood pressure, fasting glucose, total cholesterol, smoking behavior, and alcohol consumption. We additionally excluded individuals who had developed MD before the relevant CKD index date and those who died before that date.

Participants were 40–79 years of age at enrollment in the health-screening cohort and had undergone national health screening during 2002–2003. Because CKD could occur later during follow-up, some participants were older than 79 years at the CKD index date. After application of the eligibility criteria, 15,943 participants constituted the CKD cohort.

The comparison cohort was selected from 497,388 individuals who had no CKD-related diagnostic codes throughout the observation period. Each CKD participant was matched with four individuals without CKD according to age, sex, household income category, and residential region. Controls were assigned the same index date as their corresponding CKD participant. Individuals who had died or developed MD before their assigned index date were not eligible to serve as controls. A total of 417,900 potential controls were removed during the matching and eligibility process. Because control eligibility required absence of CKD codes beyond the index date, post-index information contributed to control selection.

**Figure 1:**
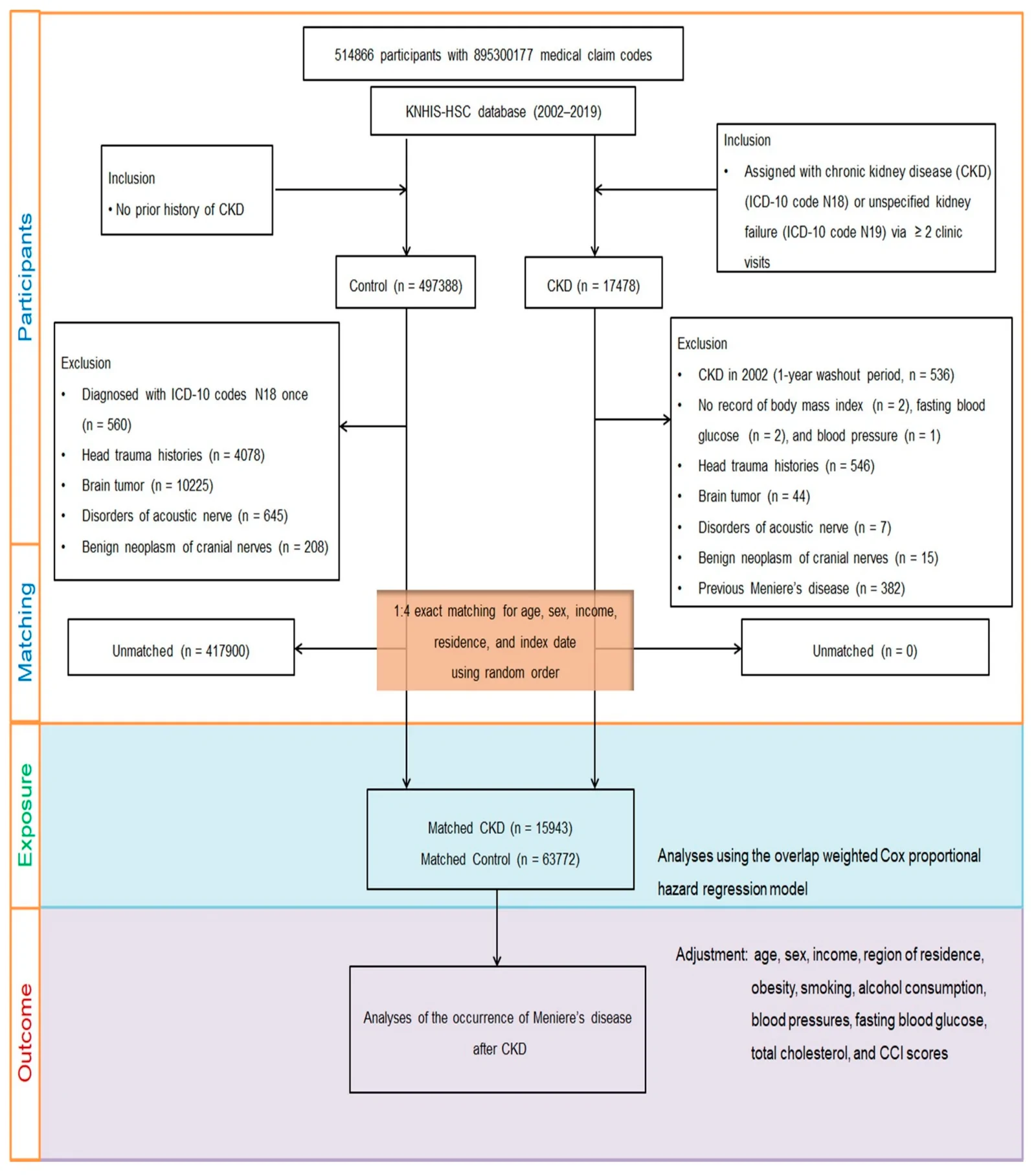
A schematic representation of the participant selection process used in this study. Out of a total of 514,866 participants, 15,943 participants with chronic kidney disease (CKD) were matched with 63,772 control participants based on age, sex, income level, and region of residence. Abbreviations: KNHIS-HSC, Health Screening Cohort of the Korean National Health Insurance Service; CCI, Charlson Comorbidity Index; ICD-10, International Classification of Diseases, 10th Revision.

The resulting cohort consisted of 15,943 participants with CKD and 63,772 matched participants without CKD. The individual participant was the experimental and analytical unit: each person contributed one exposure classification, one index date, and one follow-up record to the time-to-event analysis. No image-, field-, section-, or animal-level observations were treated as independent data points. Follow-up began on the individual index date and continued until the first occurrence of MD, death, or 31 December 2019. The participant selection process is summarized in Figure 1. Because all eligible CKD participants and matched controls available in the database were included, no separate a priori sample-size calculation was performed.

### 2.4. Covariates

Demographic, socioeconomic, behavioral, and metabolic characteristics were obtained primarily from standardized national health-screening records. Age was grouped into 10 categories, while household income was categorized into five strata. Residence was classified as urban or rural. The seven major Korean metropolitan cities, each with a population exceeding one million, were designated as urban areas, whereas the remaining administrative regions were categorized as rural.

Health-related behaviors included smoking status and frequency of alcohol consumption. Body mass index (BMI) was calculated from standardized health-screening measurements and categorized according to Asia-Pacific criteria as underweight (<18.5 kg/m^2^), normal weight (18.5–22.9 kg/m^2^), overweight (23.0–24.9 kg/m^2^), obese class I (25.0–29.9 kg/m^2^), and obese class II (≥30.0 kg/m^2^) [25]. Systolic and diastolic blood pressure, fasting serum glucose, and total cholesterol were incorporated as metabolic and cardiovascular indicators because these factors may be related to both renal disease and inner-ear disorders [26,27]. The overall burden of coexisting disease was summarized using the Charlson Comorbidity Index (CCI) [28]. The CCI assigns weighted values to 17 groups of chronic disorders and produces an aggregate comorbidity score, with higher values indicating greater disease burden. Because CKD represented the exposure under investigation, renal disease was omitted from calculation of the CCI in the present analysis to avoid adjusting for a component of the exposure itself [29].

### 2.5. Statistical Analysis

Categorical characteristics are reported as frequencies and percentages, whereas continuous variables are summarized as means with standard deviations. Baseline comparability between the CKD and non-CKD groups was evaluated using absolute standardized differences rather than conventional significance tests [30]. An absolute standardized difference < 0.10 was considered indicative of acceptable balance [30].

Propensity-score overlap weighting was used to improve covariate comparability between the CKD and comparison cohorts [31]. Individual propensity scores were estimated using multivariable logistic regression incorporating baseline demographic, socioeconomic, behavioral, metabolic, comorbidity, and prior vestibular-disorder variables. Incident MD, the study outcome, was not included in the propensity-score model. Participants with CKD were assigned a weight of 1 minus the estimated propensity score, whereas participants without CKD were assigned a weight equal to the propensity score [31,32]. This weighting approach emphasizes individuals with substantial probability of belonging to either exposure group and reduces the influence of observations with extreme propensity scores [31,32]. Covariate balance was reassessed after weighting. Variance estimates for the weighted Cox models were obtained using the model-based variance estimator.

Incidence rates were calculated as the number of newly identified MD cases divided by accumulated person-time and are expressed per 1000 person–years. Differences in unadjusted cumulative MD occurrence according to CKD status were visualized with Kaplan–Meier curves and evaluated using the log-rank test.

The primary association between CKD and incident MD was estimated using Cox proportional hazards regression. Hazard ratios (HRs) and 95% confidence intervals (CIs) were calculated before and after overlap weighting. Death was treated as a censoring event in the original analysis rather than as a competing event. The proportional hazards assumption was assessed graphically using log-minus-log survival plots.

Potential variation in the CKD–MD association was explored in subgroup analyses according to age (<70 and ≥70 years), sex, BMI category, comorbidity burden, smoking, alcohol consumption, socioeconomic characteristics, and previous vestibular disorders. These analyses were exploratory, and no adjustment for multiple comparisons was applied. Therefore, subgroup *p*-values were interpreted as nominal and hypothesis-generating rather than confirmatory. All tests were two-sided, and a *p* value < 0.05 was considered nominally statistically significant. Analyses were performed using SAS version 9.4 (SAS Institute Inc., Cary, NC, USA).

## 3. Results

### 3.1. Study Population and Covariate Balance

The final cohort consisted of 79,715 participants, including 15,943 with CKD and 63,772 matched controls. Before overlap weighting, the CKD group had a less favorable metabolic and comorbidity profile than the comparison group. In particular, differences were evident in fasting glucose, blood pressure, BMI distribution, and CCI.

Overlap weighting produced close balance in the weighted means and proportions of the measured baseline covariates, with standardized differences approaching zero (Table 1). Because the originally generated post-weighting standard deviations for continuous variables, Table 1 reports the weighted means for these continuous covariates and uses standardized differences as the prespecified measure of balance.

### 3.2. CKD and Subsequent Ménière’s Disease

During follow-up, 1076 participants developed MD, including 173 participants with CKD and 903 controls. The CKD group accumulated 67,471 person–years of observation, corresponding to an MD incidence rate of 2.56 per 1000 person–years. The comparison group accumulated 330,701 person–years, with an incidence rate of 2.73 per 1000 person–years.

The crude Cox model yielded an HR of 0.92 (95% CI, 0.79–1.09; *p* = 0.346). The original overlap-weighted analysis yielded a point estimate of 0.95 (95% CI, 0.84–1.07; *p* = 0.414) (Table 2).

**Figure 2:**
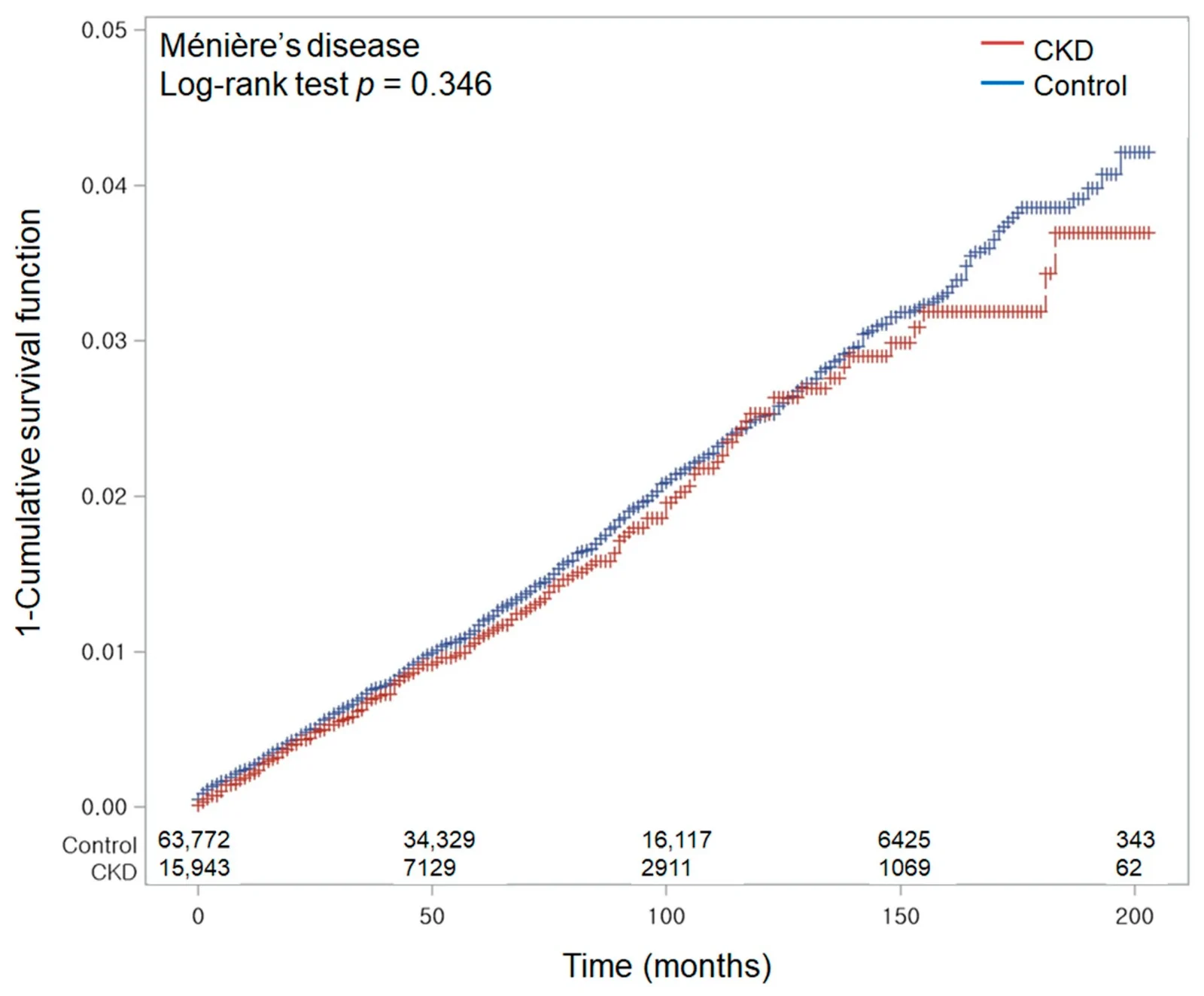
Cumulative incidence of Ménière’s disease in CKD and control groups during the 2002–2019 database observation window. Cumulative incidence was compared using the Kaplan–Meier method with the log-rank test. The analysis revealed no significant difference in the probability of developing Ménière’s disease between participants with CKD and those without CKD. Statistical significance was defined as *p* < 0.05.

Kaplan–Meier analysis showed similar cumulative MD occurrence in participants with and without CKD, with no statistically significant difference on the log-rank test (*p* = 0.346; Figure 2). Graphical assessment of the proportional hazards assumption using log-minus-log plots showed broadly parallel curves during early and middle follow-up, with some convergence in later follow-up (Supplementary Figure S1).

### 3.3. Findings Across Participant Subgroups

The overall point estimates were generally near unity across demographic strata. Among participants aged <70 years, the crude HR was 0.90 (95% CI, 0.72–1.12; *p* = 0.348) and the overlap-weighted HR was 0.89 (95% CI, 0.75–1.05; nominal *p* = 0.161). Among participants aged ≥70 years, the corresponding estimates were 0.97 (95% CI, 0.77–1.23; *p* = 0.800) and 1.01 (95% CI, 0.84–1.21; nominal *p* = 0.910), respectively.

Several exploratory subgroup estimates were below unity. Among rural residents, CKD was associated with a lower incidence of MD (overlap-weighted HR, 0.84; 95% CI, 0.71–0.99; nominal *p* = 0.038), reaching nominal statistical significance. No comparable association was observed among urban residents (overlap-weighted HR, 1.11; 95% CI, 0.93–1.34; nominal *p* = 0.255) (Table 2).

Nominal *p*-values < 0.05 were also observed among participants with CCI ≥2 (overlap-weighted HR, 0.80; 95% CI, 0.64–1.00; *p* = 0.048) and those with a history of vestibular neuronitis (overlap-weighted HR, 0.68; 95% CI, 0.49–0.95; *p* = 0.023). No comparable association was observed for benign paroxysmal positional vertigo or other peripheral vestibular disorders (Table 3). No correction for multiple comparisons was applied across these subgroup analyses. In addition, the weighted variance estimates were not robust/sandwich-based.

## 4. Discussion

In this Korean health-screening cohort derived from 16 years of nationwide longitudinal data, the crude and overlap-weighted point estimates did not suggest a higher hazard of incident MD among participants with CKD. In exploratory subgroup analyses, nominally significant inverse associations were observed among rural residents, participants in the overweight BMI category (23.0–24.9 kg/m^2^), participants with CCI scores ≥ 2, and those with a history of vestibular neuronitis. Because no adjustment for multiple comparisons was applied and the weighted analyses used model-based rather than robust/sandwich variance estimation, these subgroup findings should be interpreted cautiously as hypothesis-generating rather than as evidence of protective effects. Overall, the results do not provide definitive evidence that CKD independently increases or decreases the risk of MD.

This finding is clinically relevant because renal dysfunction has repeatedly been linked to auditory impairment. CKD can produce endothelial dysfunction, microvascular abnormalities, inflammation, oxidative stress, altered electrolyte handling, and accumulation of uremic metabolites [6,8,9,10,11,12], all of which could plausibly affect inner-ear function. Furthermore, the kidney and cochlea share several physiological features related to epithelial ion transport and microvascular homeostasis [11,12,16]. These observations have contributed to the concept of a kidney–inner ear relationship. Our findings should be considered alongside the limited epidemiologic evidence specifically examining MD in patients with CKD.

Our results indicate, however, that this relationship should not necessarily be generalized to MD. Previous studies have demonstrated associations of CKD with hearing loss and tinnitus [11,17,18,19,20,33,34], but these outcomes may differ fundamentally from MD. Hearing impairment and tinnitus are relatively nonspecific clinical manifestations and may result from vascular disease, aging, noise exposure, medication toxicity, metabolic abnormalities, or direct cochlear injury [14,35]. MD, in contrast, is a distinct audiovestibular syndrome characterized by recurrent vertigo together with characteristic auditory manifestations and is believed to involve complex disturbances of endolymph regulation as well as immune, genetic, and epithelial mechanisms [1,17,18,24,34,36,37].

The present findings also add to the limited population-based evidence directly examining CKD and MD. A previous Korean nationwide cohort study covering 2002–2013 reported an increased risk of MD among patients with CKD, although the association was substantially attenuated after multivariable adjustment [17]. Our study addresses the same clinical question using a different analytic framework and a longer observation period extending through 2019.

Several methodological differences may account for the apparently weaker association observed in the present study [11,17,18,19,20,33,34]. First, participants were drawn from a health-screening cohort that provided directly measured metabolic variables and information on health behaviors, permitting adjustment beyond diagnosis-based comorbidity measures. Second, matching was performed at a 1:4 ratio according to demographic and socioeconomic characteristics, followed by propensity-score overlap weighting to achieve close balance across additional measured covariates. Third, MD ascertainment required repeated diagnostic claims accompanied by audiometric examination codes rather than relying solely on an ICD-10 diagnosis [24]. These features may have reduced residual confounding and outcome misclassification.

Thus, rather than contradicting the established association between CKD and auditory dysfunction, our results may suggest that the influence of renal disease may differ according to the specific inner-ear phenotype under consideration [11,17,18,19,20,33,34]. CKD-related vascular or metabolic abnormalities may be sufficient to contribute to cochlear dysfunction manifested as hearing loss or tinnitus [14,35], but they may not independently initiate the more complex pathophysiological process necessary for clinically manifest MD [1,24,36].

Several subgroup estimates were below unity, including those for rural residents, participants in the overweight BMI category (23.0–24.9 kg/m^2^), those with CCI scores ≥2, and those with a history of vestibular neuronitis. These findings require cautious interpretation. Because the subgroup analyses were exploratory and no adjustment for multiple comparisons was applied, the nominally significant associations may reflect chance and should be regarded as hypothesis-generating rather than as evidence of protective effects.

The major strengths of this study include its nationwide longitudinal data source, large study population, and use of repeated diagnostic records together with audiometric examination claims to improve outcome specificity. The health-screening database also provided information on smoking, alcohol consumption, BMI, blood pressure, glucose, and cholesterol. Matching and overlap weighting achieved close balance in measured baseline covariate means and proportions, and previous vestibular disorders were considered because of their potential relationship with subsequent MD diagnosis.

The results must be interpreted in light of several limitations. First, diagnoses originated from administrative insurance data rather than direct review of clinical records, so some misclassification is unavoidable, and all Bárány Society criteria could not be clinically adjudicated [38]. Second, detailed measures of CKD severity, including longitudinal estimated glomerular filtration rate, albuminuria, and CKD stage, were unavailable. Third, propensity-score methods can address only measured covariates, and residual confounding remains possible [36,39]. Fourth, controls were required to remain free of CKD diagnoses throughout the observation period. Because this definition incorporates post-index information, control selection may have introduced selection bias; a preferable design would define control eligibility by CKD-free status at the index date. Fifth, death was treated as a censoring event rather than explicitly modeled as a competing event. Given the differing accumulated person-time between groups, differential mortality could have influenced the opportunity to receive an MD diagnosis and may have biased the cause-specific hazard estimate. A Fine–Gray competing-risk analysis and death counts by group could not be obtained because the original individual-level analytic dataset was no longer available after the authorized data-retention period [40]. Sixth, the original overlap-weighted Cox analysis did not use a robust/sandwich variance estimator. Because the authorized data-retention period had expired, the original individual-level analytic dataset was no longer available, and we were therefore unable to re-estimate the standard errors using a robust/sandwich variance estimator. Therefore, the precision of the weighted confidence intervals may have been overestimated. Seventh, overlap weighting guarantees balance in weighted means for included covariates but not equality of weighted variances. Nevertheless, the pattern of post-weighting standard deviations in the original output could not be independently reverified after data access ended; we therefore report weighted means and standardized differences in Table 1 rather than unverified post-weighting SDs. Eighth, no multiplicity adjustment was applied to the subgroup analyses, so nominally significant subgroup findings are exploratory. Ninth, graphical assessment with log-minus-log plots showed broadly parallel curves during early and middle follow-up but some convergence later, suggesting that the proportional hazards assumption may not have been fully maintained over the entire observation window. The Cox HR should therefore be viewed as an overall summary across follow-up rather than a constant effect at every time point. Finally, the study population consisted of Korean adults participating in a national health-screening program, which may limit generalizability to other populations.

With these limitations in mind, the available crude and weighted point estimates were close to unity and did not indicate an obvious increase in MD hazard among participants with CKD. However, the unresolved uncertainty in weighted variance estimation, the lack of competing-risk analysis, and the control-selection strategy prevent a definitive conclusion that clinically meaningful excess risk is absent. Future studies should prospectively retain individual-level follow-up data and apply risk-set-based control selection, robust variance estimation for weighting, formal competing-risk methods, and time-varying analyses when appropriate.

## 5. Conclusions

In this nationwide health-screening cohort, the crude and overlap-weighted point estimates did not suggest a higher hazard of incident MD among participants with CKD. However, these findings should be interpreted cautiously in light of limitations in weighted variance estimation and the absence of a competing-risk analysis for death. Clinically, CKD status alone should not currently be used as a basis for MD-specific risk stratification or intensified surveillance in the absence of characteristic audiovestibular symptoms. Patients with CKD who develop vertigo, fluctuating hearing loss, tinnitus, or aural fullness should continue to undergo standard otologic and vestibular evaluation. The nominally significant inverse associations observed in selected subgroups were exploratory and should not influence clinical management without independent confirmation. Future prospective longitudinal studies incorporating serial estimated glomerular filtration rate, albuminuria, CKD stage, electrolyte profiles, medication exposure, specific renal phenotypes, competing-risk methods, and clinically adjudicated Bárány Society criteria are needed to clarify whether CKD severity or particular renal phenotypes influence MD risk.

## Figures and Tables

**Table 1:** Summary of baseline features of the participants.

Characteristics		Before Overlap Weighting Adjustment			After Overlap Weighting Adjustment		
		CKD	Control	Standardized Difference	CKD	Control	Standardized Difference
Age (years), *n* (%)				0.00			0.00
	40–44	94 (0.59)	376 (0.59)		72 (0.61)	72 (0.61)	
	45–49	354 (2.22)	1416 (2.22)		256 (2.17)	256 (2.17)	
	50–54	915 (5.74)	3660 (5.74)		664 (5.62)	664 (5.62)	
	55–59	1785 (11.20)	7140 (11.20)		1304 (11.04)	1304 (11.04)	
	60–64	2191 (13.74)	8764 (13.74)		1599 (13.54)	1599 (13.54)	
	65–69	2471 (15.50)	9884 (15.50)		1813 (15.35)	1813 (15.35)	
	70–74	2805 (17.59)	11,220 (17.59)		2089 (17.69)	2089 (17.69)	
	75–79	2708 (16.99)	10,832 (16.99)		2026 (17.16)	2026 (17.16)	
	80–84	1767 (11.08)	7068 (11.08)		1330 (11.27)	1330 (11.27)	
	85+	853 (5.35)	3412 (5.35)		654 (5.54)	654 (5.54)	
Sex (*n*, %)				0.00			0.00
	Male	10,521 (65.99)	42,084 (65.99)		7800 (66.06)	7800 (66.06)	
	Female	5422 (34.01)	21,688 (34.01)		4007 (33.94)	4007 (33.94)	
Income (*n*, %)				0.00			0.00
	1 (lowest)	2775 (17.41)	11,100 (17.41)		2047 (17.34)	2047 (17.34)	
	2	1850 (11.60)	7400 (11.60)		1370 (11.60)	1370 (11.60)	
	3	2263 (14.19)	9052 (14.19)		1670 (14.14)	1670 (14.14)	
	4	3176 (19.92)	12,704 (19.92)		2342 (19.83)	2342 (19.83)	
	5 (highest)	5879 (36.88)	23,516 (36.88)		4378 (37.08)	4378 (37.08)	
Region of residence (*n*, %)				0.00			0.00
	Urban	6870 (43.09)	27,480 (43.09)		5085 (43.06)	5085 (43.06)	
	Rural	9073 (56.91)	36,292 (56.91)		6722 (56.94)	6722 (56.94)	
Weight status (*n*, %)				0.16			0.00
	Underweight	419 (2.63)	2143 (3.36)		330 (2.79)	330 (2.79)	
	Normal	4900 (30.73)	22,793 (35.74)		3744 (31.71)	3744 (31.71)	
	Overweight	4161 (26.10)	17,024 (26.70)		3108 (26.32)	3108 (26.32)	
	Obese I	5736 (35.98)	20,002 (31.36)		4149 (35.14)	4149 (35.14)	
	Obese II	727 (4.56)	1810 (2.84)		476 (4.03)	476 (4.03)	
Smoking status (*n*, %)				0.02			0.00
	Non-smoker	10,148 (63.65)	41,060 (64.39)		7542 (63.87)	7542 (63.87)	
	Past smoker	1670 (10.47)	6781 (10.63)		1251 (10.60)	1251 (10.60)	
	Current smoker	4125 (25.87)	15,931 (24.98)		3014 (25.53)	3014 (25.53)	
Alcohol consumption (*n*, %)				0.07			0.00
	<1 time a week	11,569 (72.56)	44,352 (69.55)		8475 (71.78)	8475 (71.78)	
	≥1 time a week	4374 (27.44)	19,420 (30.45)		3332 (28.22)	3332 (28.22)	
SBP, mmHg, Mean ± SD		131.82 ± 18.43	128.71 ± 16.29	0.18	130.86	130.86	0.00
DBP, mmHg, Mean ± SD		78.74 ± 11.51	78.12 ± 10.37	0.06	78.53	78.53	0.00
Fasting blood glucose, mg/dL, Mean ± SD		115.59 ± 49.23	103.71 ± 28.61	0.30	109.98	109.98	0.00
Total cholesterol, mg/dL, Mean ± SD		190.49 ± 45.63	193.33 ± 38.67	0.07	190.86	190.86	0.00
CCI score, Mean ± SD		2.16 ± 2.20	1.10 ± 1.71	0.54	1.81	1.81	0.00
Benign paroxysmal vertigo history (*n*, %)		2365 (14.83)	9373 (14.70)	0.00	1767 (14.97)	1767 (14.97)	0.00
Vestibular neuronitis history (*n*, %)		658 (4.13)	2513 (3.94)	0.01	484 (4.10)	484 (4.10)	0.00
Other peripheral vestibular disorders history (*n*, %)		2022 (12.68)	7697 (12.07)	0.02	1497 (12.68)	1497 (12.68)	0.00

Abbreviations: CCI, Charlson Comorbidity Index; SBP, systolic blood pressure; DBP, diastolic blood pressure; CKD, chronic kidney disease; SD, standard deviation. For continuous variables, unweighted values are presented as mean (SD), whereas the post-overlap-weighting columns report weighted means. Standardized differences were used to assess covariate balance.

**Table 2:** Crude and overlap propensity score weighted hazard ratios (95% confidence interval) of CKD for Ménière’s disease with subgroup analyses according to age, sex, income, and region of residence.

		N of Event/N of Total (%)	Follow-Up Duration (PY)	IR per 1000 (PY)	Hazard Ratios for Ménière’s Disease			
					Crude	*p*-Value	Overlap Weighted Model †	*p*-Value
Total participants								
	CKD	173/15,943 (1.09)	67,471	2.56	0.92 (0.79–1.09)	0.346	0.95 (0.84–1.07)	0.414
	Control	903/63,772 (1.42)	330,701	2.73	1		1	
Age < 70 years								
	CKD	91/7810 (1.17)	43,594	2.09	0.90 (0.72–1.12)	0.348	0.89 (0.75–1.05)	0.161
	Control	482/31,240 (1.54)	206,557	2.33	1		1	
Age ≥ 70 years								
	CKD	82/8133 (1.01)	23,877	3.43	0.97 (0.77–1.23)	0.80	1.01 (0.84–1.21)	0.910
	Control	421/32,532 (1.29)	124,144	3.39	1		1	
Male								
	CKD	96/10,521 (0.91)	43,493	2.21	0.88 (0.71–1.10)	0.262	0.91 (0.77–1.07)	0.248
	Control	522/42,084 (1.24)	212,709	2.45	1		1	
Female								
	CKD	77/5422 (1.42)	23,978	3.21	0.98 (0.77–1.25)	0.884	0.99 (0.83–1.20)	0.957
	Control	381/21,688 (1.76)	117,992	3.23	1		1	
Low-income group								
	CKD	73/6888 (1.06)	28,722	2.54	0.96 (0.75–1.24)	0.762	0.94 (0.78–1.14)	0.549
	Control	374/27,552 (1.36)	143,813	2.60	1		1	
High-income group								
	CKD	100/9055 (1.10)	38,749	2.58	0.90 (0.73–1.11)	0.327	0.95 (0.81–1.11)	0.504
	Control	529/36,220 (1.46)	186,888	2.83	1		1	
Urban resident								
	CKD	83/6870 (1.21)	30,776	2.70	1.07 (0.84–1.36)	0.568	1.11 (0.93–1.34)	0.255
	Control	369/27,480 (1.34)	148,330	2.49	1		1	
Rural resident								
	CKD	90/9073 (0.99)	36,695	2.45	0.82 (0.66–1.03)	0.086	0.84 (0.71–0.99)	0.038 *
	Control	534/36,292 (1.47)	182,371	2.93	1		1	

Abbreviations: CKD, chronic kidney disease; IR, incidence rate; PY, person–year. * Nominal *p* < 0.05; no adjustment for multiple comparisons was applied. Subgroup findings are exploratory. † Adjusted for age, sex, income, region of residence, obesity, smoking, alcohol consumption, systolic blood pressure, diastolic blood pressure, fasting blood glucose, total cholesterol, and Charlson Comorbidity Index scores. The overlap-weighted confidence intervals and *p*-values were calculated using model-based variance estimates without a robust/sandwich variance estimator and may therefore overstate precision.

**Table 3:** Subgroup analyses of crude and overlap propensity score weighted hazard ratios (95% confidence interval) of CKD for Ménière’s disease.

		N of Event/N of Total (%)	Follow-Up Duration (PY)	IR per 1000 (PY)	Hazard Ratios for Ménière’s Disease			
					Crude	*p*-Value	Overlap Weighted Model †	*p*-Value
Underweight								
	CKD	5/419 (1.19)	1307	3.83	2.33 (0.84–6.42)	0.103	2.39 (0.98–5.83)	0.055
	Control	15/2143 (0.70)	9401	1.60	1		1	
Normal weight								
	CKD	52/4900 (1.06)	19,945	2.61	1.04 (0.78–1.40)	0.782	1.09 (0.87–1.35)	0.461
	Control	289/22,793 (1.27)	117,952	2.45	1		1	
Overweight								
	CKD	40/4161 (0.96)	18,690	2.14	0.67 (0.48–0.94)	0.02 *	0.68 (0.54–0.87)	0.002 *
	Control	282/17,024 (1.66)	90,226	3.13	1		1	
Obese								
	CKD	76/6463 (1.18)	27,529	2.76	0.98 (0.76–1.26)	0.866	1.01 (0.82–1.23)	0.954
	Control	317/21,812 (1.45)	113,122	2.80	1		1	
Non-smoker								
	CKD	127/10,148 (1.25)	44,446	2.86	0.93 (0.77–1.13)	0.457	0.96 (0.83–1.11)	0.566
	Control	658/41,060 (1.60)	217,147	3.03	1		1	
Past and current smoker								
	CKD	46/5795 (0.79)	23,025	2.00	0.91 (0.66–1.24)	0.537	0.91 (0.72–1.16)	0.466
	Control	245/22,712 (1.08)	113,554	2.16	1		1	
Alcohol consumption <1 time a week								
	CKD	132/11,569 (1.14)	49,865	2.65	0.89 (0.74–1.08)	0.232	0.93 (0.81–1.07)	0.322
	Control	679/44,352 (1.53)	232,391	2.92	1		1	
Alcohol consumption ≥1 time a week								
	CKD	41/4374 (0.94)	17,606	2.33	1.00 (0.72–1.40)	0.978	1.02 (0.79–1.31)	0.898
	Control	224/19,420 (1.15)	98,310	2.28	1		1	
SBP < 140 mmHg and DBP < 90 mmHg								
	CKD	120/10,455 (1.15)	42,645	2.81	0.99 (0.81–1.20)	0.911	0.97 (0.84–1.12)	0.697
	Control	657/46,340 (1.42)	234,663	2.80	1		1	
SBP ≥ 140 mmHg or DBP ≥ 90 mmHg								
	CKD	53/5488 (0.97)	24,826	2.13	0.82 (0.61–1.11)	0.199	0.89 (0.70–1.13)	0.352
	Control	246/17,432 (1.41)	96,038	2.56	1		1	
Fasting blood glucose < 100 mg/dL								
	CKD	95/7453 (1.27)	34,180	2.78	0.97 (0.78–1.20)	0.748	0.96 (0.82–1.12)	0.595
	Control	563/35,449 (1.59)	198,134	2.84	1		1	
Fasting blood glucose ≥ 100 mg/dL								
	CKD	78/8490 (0.92)	33,291	2.34	0.90 (0.70–1.15)	0.404	0.94 (0.77–1.15)	0.540
	Control	340/28,323 (1.20)	132,567	2.56	1		1	
Total cholesterol < 200 mg/dL								
	CKD	97/9853 (0.98)	38,707	2.51	0.88 (0.71–1.09)	0.249	0.89 (0.76–1.06)	0.186
	Control	515/37,390 (1.38)	184,295	2.79	1		1	
Total cholesterol ≥ 200 mg/dL								
	CKD	76/6090 (1.25)	28,764	2.64	0.99 (0.77–1.26)	0.919	1.05 (0.87–1.26)	0.626
	Control	388/26,382 (1.47)	146,406	2.65	1		1	
CCI scores = 0								
	CKD	66/4745 (1.39)	22,212	2.97	1.17 (0.90–1.51)	0.243	1.10 (0.93–1.30)	0.283
	Control	467/34,875 (1.34)	185,059	2.52	1		1	
CCI scores = 1								
	CKD	30/2703 (1.11)	10,919	2.75	0.95 (0.64–1.40)	0.782	1.02 (0.76–1.37)	0.893
	Control	171/11,430 (1.50)	59,317	2.88	1		1	
CCI scores ≥ 2								
	CKD	77/8495 (0.91)	34,340	2.24	0.71 (0.55–0.92)	0.009 *	0.80 (0.64–1.00)	0.048 *
	Control	265/17,467 (1.52)	86,325	3.07	1		1	
Non-Benign paroxysmal vertigo history								
	CKD	86/13,578 (0.63)	57,042	1.51	0.84 (0.67–1.06)	0.137	0.88 (0.74–1.05)	0.149
	Control	498/54,399 (0.92)	281,723	1.77	1		1	
Benign paroxysmal vertigo history								
	CKD	87/2365 (3.68)	10,429	8.34	1.00 (0.79–1.26)	0.974	1.03 (0.87–1.24)	0.709
	Control	405/9373 (4.32)	48,978	8.27	1		1	
Non-vestibular neuronitis history								
	CKD	150/15,285 (0.98)	64,550	2.32	0.97 (0.82–1.16)	0.761	1.02 (0.89–1.16)	0.816
	Control	749/61,259 (1.22)	317,534	2.36	1		1	
Vestibular neuronitis history								
	CKD	23/658 (3.50)	2921	7.87	0.65 (0.42–1.01)	0.055	0.68 (0.49–0.95)	0.023 *
	Control	154/2513 (6.13)	13,167	11.70	1		1	
Non-other peripheral vestibular disorders history								
	CKD	113/13,921 (0.81)	58,515	1.93	0.97 (0.80–1.19)	0.804	1.04 (0.89–1.21)	0.612
	Control	568/56,075 (1.01)	289,973	1.96	1		1	
Other peripheral vestibular disorders history								
	CKD	60/2022 (2.97)	8956	6.70	0.80 (0.61–1.05)	0.105	0.81 (0.66–1.00)	0.053
	Control	335/7697 (4.35)	40,728	8.23	1		1	

Abbreviations: CKD, chronic kidney disease; IR, incidence rate; PY, person–year. * Nominal *p* < 0.05; no adjustment for multiple comparisons was applied. Subgroup findings are exploratory. † Adjusted for age, sex, income, region of residence, obesity, smoking, alcohol consumption, systolic blood pressure, diastolic blood pressure, fasting blood glucose, total cholesterol, Charlson Comorbidity Index scores, and prior vestibular disorders. The overlap-weighted confidence intervals and *p*-values were calculated using model-based variance estimates without a robust/sandwich variance estimator and may therefore overstate precision.

## Data Availability

All data are available from the database of National Health Insurance Sharing Service (NHISS) https://nhiss.nhis.or.kr/ (accessed on 1 November 2024). The NHISS allows access to all of this data for any researcher who agrees to follow research ethics for a processing charge. If you want to access the data of this article, you can download it from the website after agreeing to follow the research ethics.

## Supplementary Materials

The following supporting information can be downloaded at https://www.mdpi.com/article/10.3390/jcm15187328/s1. Figure S1. Log-minus-log plots of estimated survival functions according to CKD status. The control and CKD curves are broadly parallel during the early and middle portions of follow-up, with some convergence during later follow-up. Thus, the proportional hazards assumption appears approximately reasonable over much of the observation period, although some time-varying departure cannot be excluded at later times. Accordingly, the Cox hazard ratio is interpreted as an overall summary association across follow-up rather than as a strictly constant effect at every time point.

## Abbreviations

CKD	Chronic kidney disease
MD	Ménière’s disease
BMI	Body mass index
ICD-10	International Classification of Diseases, 10th Revision
HR	Hazard ratio
CI	Confidence interval
CCI	Charlson Comorbidity Index
IR	Incidence rate
PY	Person–year
