# Supplementary material for: Association Between Chronic Kidney Disease and Incident Ménière’s Disease Using 16 Years of Nationwide Longitudinal Data
Source: J Clin Med. 2026 Sep 21;15(18):7328. doi: 10.3390/jcm15187328 (PMC13607501; doi:10.3390/jcm15187328)
Supplement: Supplementary file 1 [file jcm-15-07328-s001.zip › jcm-4550750-supplementary.pdf]

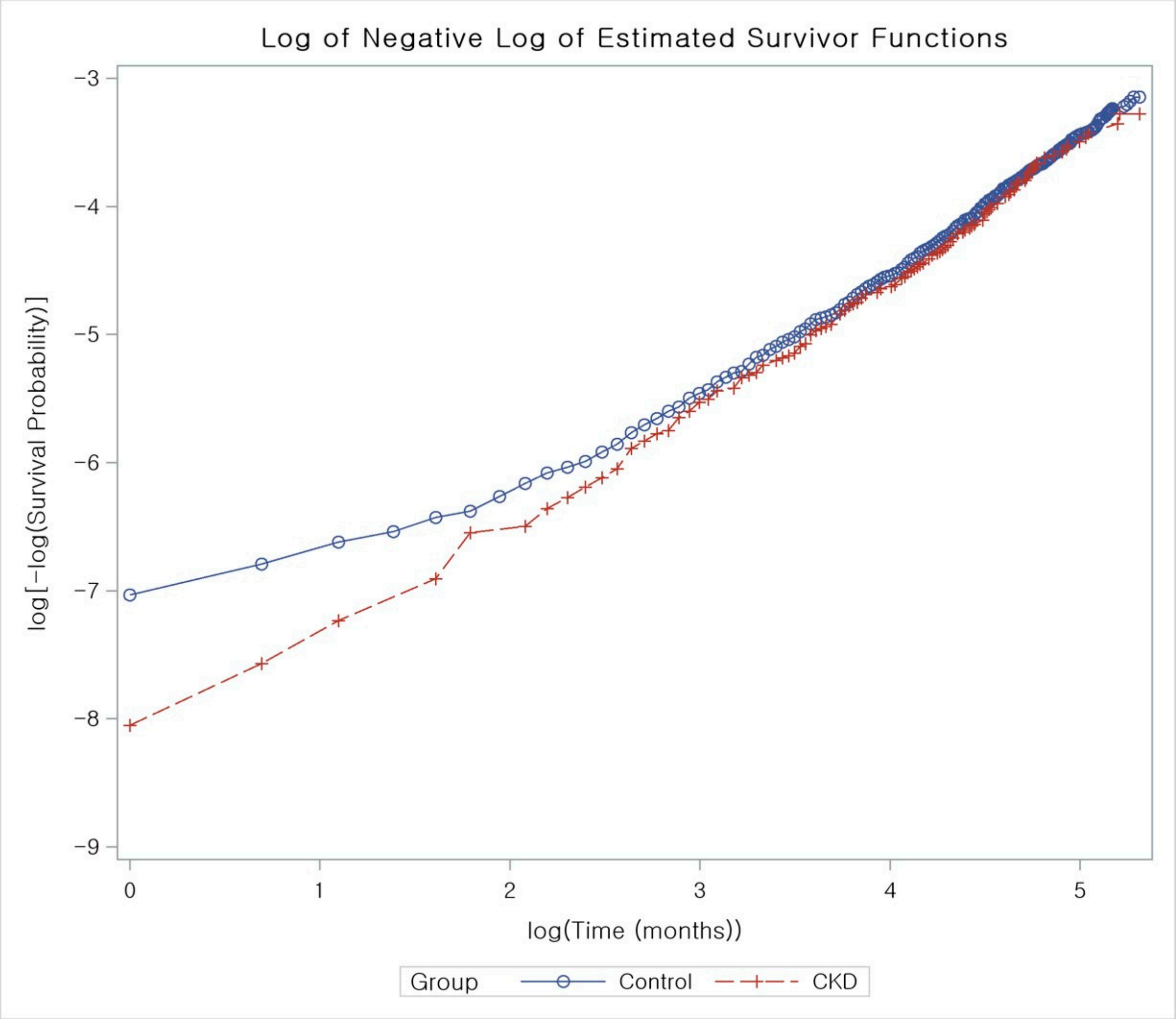

Figure S1. Log-minus-log plots of estimated survival functions according to CKD status. The control and CKD curves are broadly parallel during the early and middle portions of follow-up, with some convergence during later follow-up. Thus, the proportional hazards assumption appears approximately reasonable over much of the observation period, although some time-varying departure cannot be excluded at later times. Accordingly, the Cox hazard ratio is interpreted as an overall summary association across follow-up rather than as a strictly constant effect at every time point.
